# Rapid Development of Non-Alcoholic Steatohepatitis in *Psammomys obesus* (Israeli Sand Rat)

**DOI:** 10.1371/journal.pone.0092656

**Published:** 2014-03-20

**Authors:** Briana Spolding, Timothy Connor, Carrie Wittmer, Lelia L. F. Abreu, Antony Kaspi, Mark Ziemann, Gunveen Kaur, Adrian Cooper, Shona Morrison, Scott Lee, Andrew Sinclair, Yann Gibert, James L. Trevaskis, Jonathon D. Roth, Assam El-Osta, Richard Standish, Ken Walder

**Affiliations:** 1 Metabolic Research Unit, School of Medicine, Deakin University, Geelong, Victoria, Australia; 2 Amylin Pharmaceuticals, LLC., San Diego, California, United States of America; 3 Baker IDI Heart and Diabetes Institute, Melbourne, Victoria, Australia; 4 Institute of Sport, Exercise and Active Living (ISEAL), Victoria University, Melbourne, Victoria, Australia; Clermont Université, France

## Abstract

**Background and Aims:**

A major impediment to establishing new treatments for non-alcoholic steatohepatitis is the lack of suitable animal models that accurately mimic the biochemical and metabolic characteristics of the disease. The aim of this study was to explore a unique polygenic animal model of metabolic disease as a model of non-alcoholic steatohepatitis by determining the effects of 2% dietary cholesterol supplementation on metabolic and liver endpoints in *Psammomys obesus* (Israeli sand rat).

**Methods:**

*P. obesus* were provided *ad libitum* access to either a standard rodent diet (20% kcal/fat) or a standard rodent diet supplemented with 2% cholesterol (w/w) for 4 weeks. Histological sections of liver from animals on both diets were examined for key features of non-alcoholic steatohepatitis. The expression levels of key genes involved in hepatic lipid metabolism were measured by real-time PCR.

**Results:**

*P. obesus* fed a cholesterol-supplemented diet exhibited profound hepatomegaly and steatosis, and higher plasma transaminase levels. Histological analysis identified extensive steatosis, inflammation, hepatocyte injury and fibrosis. Hepatic gene expression profiling revealed decreased expression of genes involved in delivery and uptake of lipids, and fatty acid and triglyceride synthesis, and increased expression of genes involved in very low density lipoprotein cholesterol synthesis, triglyceride and cholesterol export.

**Conclusions:**

*P. obesus* rapidly develop non-alcoholic steatohepatitis when fed a cholesterol-supplemented diet that appears to be histologically and mechanistically similar to patients.

## Introduction

Non-alcoholic fatty liver disease (NAFLD) is the world's most common form of chronic liver disease, and affects approximately 75% of patients with obesity and type 2 diabetes [1]. The presence of NAFLD worsens outcomes in these patients, as it exacerbates diabetes, and can lead to cirrhosis and liver cancer [2]. It is estimated that approximately 20% of NAFLD patients will go on to develop non-alcoholic steatohepatitis (NASH), and approximately 20% of NASH patients will progress to life-threatening cirrhosis [3]. The presence of NASH in human patients is confirmed by key histological features including steatosis, inflammation, hepatocyte injury, and fibrosis [4], [5].

Current management of NASH generally focuses on weight loss and treatment of co-morbidities (such as insulin resistance [6]). A major impediment to establishing and characterising new treatments for NASH is the lack of suitable animal models that accurately mimic the biochemical and metabolic characteristics of NASH in human patients [7]. Current animal models of NAFLD or NASH can be broadly categorised as either dietary-induced or genetic. The most widely used dietary-based model is rodents fed a methionine- and choline-deficient (MCD) diet high in sucrose and fat, resulting in the prevention of hepatic β-oxidation and impaired production of very low density lipoprotein (VLDL). This leads to the accumulation of intrahepatic lipid, fibrosis and decreased VLDL synthesis. However, the MCD diet is associated with considerable weight loss and toxicity [7], [8]. Additionally, the metabolic profile of animals fed this diet includes decreased plasma insulin and glucose levels, in contrast to the metabolic profile observed in the majority of human patients with NAFLD/NASH [7], [8]. *Db/db* mice, which have a mutated leptin receptor gene, are a widely used genetic model of NAFLD. These animals develop obesity, insulin resistance, diabetes and hepatic steatosis, however only progress to NASH following additional intervention such as exposure to the MCD diet [9]. Although some of these animal models are useful for investigation into particular aspects of human NAFLD/NASH, none display all of the histological characteristics present in the human disease state and in the context of metabolic disease.

*Psammomys obesus* (Israeli sand rat) is a gerbil that is a unique animal model of the Metabolic Syndrome. In its native desert environment, *P. obesus* remain lean and healthy. However, when housed under laboratory conditions and fed a standard rodent diet (20% kcal/fat), a proportion of *P. obesus* become obese and type 2 diabetic, while others remain lean and healthy [10]–[14]. Previous studies have shown *P. obesus* to be susceptible to steatosis when fed a standard rodent diet with added fibre (30% wheat straw), however key features of human NASH such as fibrosis, hepatocyte injury and inflammation were absent [15]. The majority of previously reported rodent models of NAFLD/NASH utilize diets high in fat and/or supplemented with cholesterol. As *P. obesus* will not consume highly palatable energy-rich diets, we tested whether NASH could be induced in *P. obesus* via addition of cholesterol (2% w/w) to their standard rodent diet (20% kcal/fat). In this study we show that dietary cholesterol supplementation for 4 weeks induces NASH in *P. obesus* with a profile similar to that seen in patients.

## Materials and Methods

### Ethics Statement

Animals were maintained in accordance with the Code of Practice of the National Health and Medical Research Council of Australia, and all procedures were carried out subject to the approval of the Deakin University Animal Ethics Committee (permit number A59-2010). All efforts were made to minimise animal suffering.

### Experimental animals

A colony of outbred *P. obesus* is maintained at Deakin University, Geelong, Australia. All animals were housed in a temperature-controlled room (22±1°C) with a 12–12 h light-dark cycle (light 06:00–18:00 h).

Male animals aged 8–10 weeks were randomly allocated to the following groups: 1) Standard diet (20% kcal/fat, 0% cholesterol; n = 8), or 2) Cholesterol-supplemented diet (20% kcal/fat, 2% cholesterol (w/w); n = 9). Both diets contained 16% kcal/protein and 64% kcal/carbohydrate, and had total digestible energy of 15 MJ/kg. The source of fat for both diets was mixed vegetable oils. There was no difference in % fatty acid profile or total digestible energy between the two diets. All animals were allowed to consume their respective diet *ad libitum* for 4 weeks. Food intake was measured weekly by rate of disappearance, and body weight was measured weekly.

### Histological and biochemical analyses of liver tissue

Animals were humanely killed and 1 g of liver tissue (left lobe) was removed and fixed in 10% neutral-buffered formalin. The liver tissue was paraffin-embedded, sectioned (5 μM), mounted and stained with hematoxylin and eosin, or Masson trichrome stain (Sigma-Aldrich, St Louis, MO) to visualise fibrosis. All histological analyses were conducted by a pathologist blinded to the treatment conditions.

Pictures of Masson trichrome stained liver sections were taken on an Axioskop 2 microscope (Zeiss; magnification 200x) and collagen content was quantitated using ImageJ software (NIH). For each animal, 10 fields per liver section were selected randomly and blue staining (representing collagen) was highlighted. The area of staining was measured as number of pixels per picture and the data collated to give a mean area of staining per field for each animal in the study.

An additional portion of the liver (right lobe) was used to measure lipid content. Lipid was extracted using a modified Folch protocol [16]. The tissue was homogenised in 2∶1 chloroform/methanol solution (10 ml), and filtered. An additional 5 ml of 2∶1 chloroform/methanol solution was added, followed by 2.5 ml of 0.9% NaCl. After mixing, the extract was centrifuged for 5 min at 2 000 *g* at 10°C. After discarding the aqueous layer, the organic layer was dried under nitrogen, and total lipid content assessed by weighing.

To assess the types of lipid present, a 1 g portion of the liver (right lobe) was minced and lipids extracted essentially as previously described [17]. The lipid extract was reconstituted in 200 μl of dichloromethane and the lipids were then separated by thin layer chromatography. The lipid extracts were spotted onto silica gel plates (Silica gel 60 G, Merck, Germany) and developed in 85∶15∶2 (v/v) petroleum ether: diethylether: acetic acid in paper-lined tanks. The lipids were visualised under ultraviolet light after reacting with 0.1% (w/v) 2′,7′ –dichlorofluroescein indicator in ethanol (Scharlau, Spain).

### Plasma enzyme and metabolite analyses

Blood glucose was measured on days 0 and 28 using a glucometer (Accuchek II; Roche, Castle Hill, Australia). Plasma triglyceride, total cholesterol, HDL-cholesterol, ALT and AST levels were measured using an Olympus AU400e Bioanalyzer (Olympus America Diagnostics, Center Valley, PA). Plasma insulin concentration was measured using an ultra-sensitive ELISA kit (Crystal Chem Inc, Downers Grove, IL, USA).

### RNA Extraction and Gene Expression Analysis

Total RNA was extracted from 100 mg of liver tissue using TRIzol (Invitrogen Life Technologies, Carlsbad, USA) and RNeasy columns (Qiagen, Hilden, Germany). Quality and concentration of mRNA was established using RNA 6000 Nano Assay on a Bioanalyser (Agilent Technologies, USA), and cDNA was generated from total RNA using Superscript First-Strand Synthesis System for RT-PCR (Invitrogen). Gene expression levels were measured using FastStart Universal SYBR Green Master (Roche Australia) on a Mx3005P cycler (Stratagene), and normalised to cyclophilin. RT-PCR primer sequences are listed in Table 1.

**Table 1:** PCR primer sequences.

Gene	Forward primer (5′-3′)	Reverse primer (5′-3′)
*CD68*	ggacagcttacctttggattcaa	ctgtgggaaggacacattgtattc
*IL1B*	atcagcacttcccaagcaga	agagacggattccaaggtga
*CCL2*	catagcggtcactccgacag	catgcacctgcagctaatgc
*EMR1*	gcaagatcctcagtgcgtct	gcataccagggagatgaccg
*COL1A1*	ggtgacaagggtgagacag	agagggaccttgttcacc
*ACTA2*	cttggggttcagtggtgctt	tccaatcgaacacggaatcatc
*TGFB1*	aaagccctgtatgccgtctc	cagcaacaattcctggcgtt
*SREBF2*	ctccgcagacgaggatcatccag	cagggctgccatctgtgttcag
*SCARB1*	gtctacagggagttcagaca	taggcagtacaatgtagtcac
*LDLR*	tgacgggctggcggtagact	cccaatctgtccagtacatgaagcc
*PCSK9*	ctgaagttgccccatgtggagta	ggtatctaagagatacacctccac
*FABP1*	gcccatatgaacttctccggcaagtac	ctgggatccctaaattctcttgctgactctctt
*PPARA*	tgcatgtccgtggagaccgt	cagcatcccgtctttgttcatca
*GPAM*	tgatcagccaggagcagctg	agacagtatgtggcactctc
*HSL*	cgagacaggcctcagtgtga	aactctgggtctatggcgaatc
*LIPE*	tcgggtgtggtgggtttgg	gcgtgagatgtgttgctgagg
*MTTP*	gttctcctctccttcgtcag	ccaaccttgtgtcctctcc
*ACAT2*	tagcattcctcacccaac	ccagtccatagccatagg
*ABCA1*	aaaggaggacagtgtttc	gatgaggttggagatagc
*PPARD*	gcccttcagtgacatcattgagccc	gcagcttggggaagaggtactgg
*CPT1A*	tgcaaagatcaatcggaccc	acgccactcacgatgttcttc
*CYP7A1*	ctatgatgagggctttgag	aataggaggagcattggc
*Cyclophilin*	cccaccgtgttcttcgaca	ccagtgctcagagcacgaaa

### Statistical Analysis

Data distribution was tested for normality using a Kolmogorov-Smirnov test. Group mean differences were assessed using Student's unpaired *t*-test (for normally distributed data) or Mann-Whitney U-test. Correlation between continuous variables was assessed using Pearson (for normally distributed data) or Spearman rho tests as appropriate. All analyses were performed using Statistical Package for the Social Sciences software (SPSS version 20; USA). Data were considered statistically significant at p<0.05.

## Results

We evaluated the effects of feeding lean, non-diabetic *P. obesus* a standard rodent diet (20% kcal/fat) supplemented with 2% cholesterol for 4 weeks. There was no difference in food intake between the groups (standard diet 52±3 v. cholesterol-supplemented diet 47±3 g/kg/d, p = 0.26). The mean body weight of the two groups was not different at the start or end of the study, and body weight gain in the two groups was similar (standard diet +41.0±4.1 v. cholesterol-supplemented diet +36.1±3.7 g, p = 0.40). Blood glucose levels increased marginally in the standard diet group (+0.5±0.2 mmol/L, p = 0.04) and decreased slightly in the cholesterol-supplemented group (−0.4±0.1 mmol/L, p = 0.03). However, these changes are considered minor since all animals in the study remained normoglycemic (range 3.2–5.1 mmol/L).

**Figure 1:**
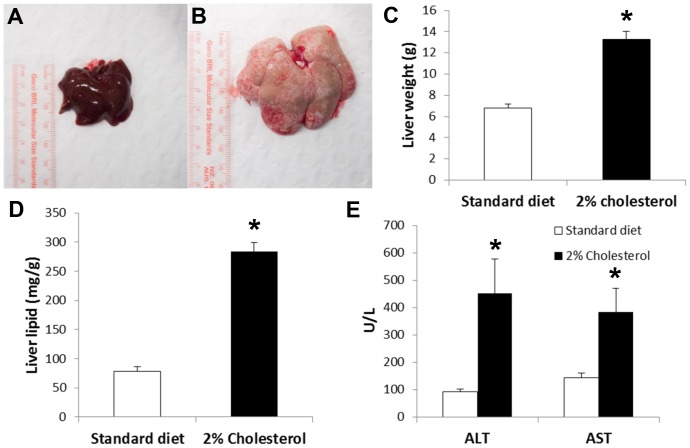
Effects of dietary cholesterol supplementation on *P. obesus* liver. A&B) Livers from *P. obesus* fed the standard diet or cholesterol-supplemented diet. C) Liver weight, *p<0.001, D) Liver lipid, *p<0.001, and E) plasma ALT and AST in *P. obesus* fed either the standard diet (20% kcal/fat, 0% cholesterol; n = 8) or cholesterol-supplemented diet (20% kcal/fat, 2% cholesterol; n = 9).

**Figure 2:**
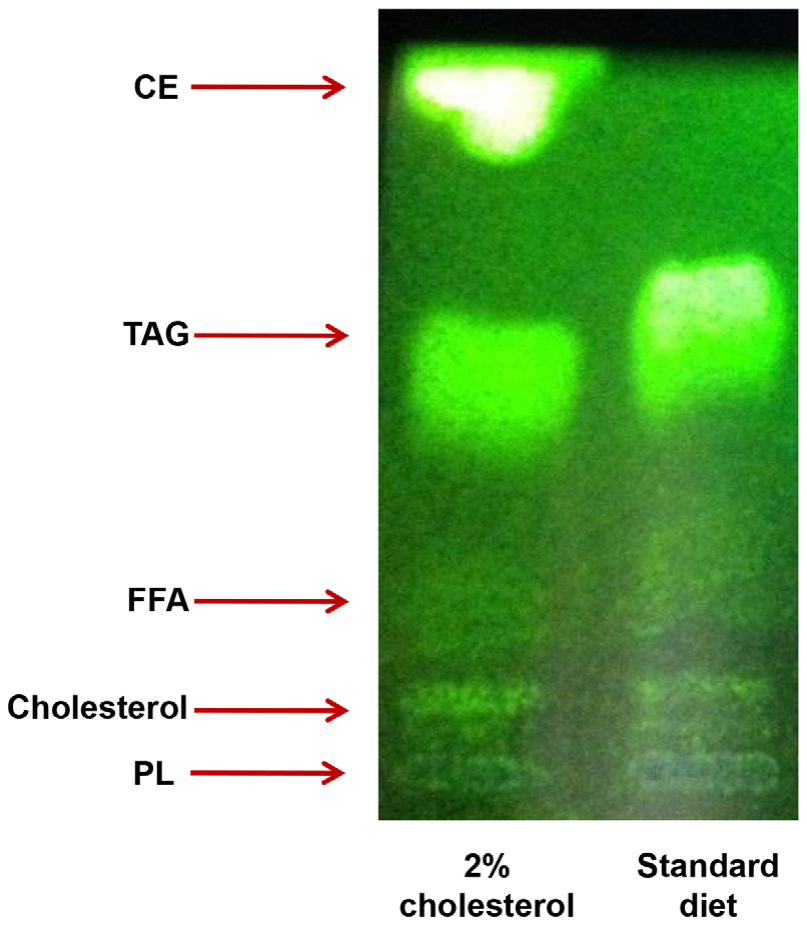
Hepatic lipid species in *P. obesus*. Thin layer chromatography analysis of hepatic lipid species in *P. obesus* fed either the standard diet or cholesterol-supplemented diet. Note that this technique is not quantitative, as equal amounts of lipid were analysed in each lane. CE = cholesterol esters, TAG = triacylglycerides, FFA = free fatty acids, PL = phospholipids.

Visual examination of livers at necropsy revealed grossly enlarged and pale livers in the animals fed the cholesterol-supplemented diet, and these livers had a firm, rubbery texture (Fig. 1A,B.). Liver weights were significantly greater in animals fed the cholesterol-supplemented diet (p<0.001, Fig. 1C.). Correspondingly, liver lipid levels were also markedly elevated (p<0.001, Fig. 1D.). Thin layer chromatography analysis showed that the lipid species present in the liver were predominantly triglycerides and, in the case of the animals fed the cholesterol-supplemented diet, cholesterol esters (Fig. 2.).

Hepatocyte injury and impaired liver function were also apparent, with plasma ALT and AST activity significantly elevated in the animals fed the cholesterol-supplemented diet (p = 0.02 and p = 0.03, respectively, Fig. 1E.). Plasma total cholesterol levels were 4-fold higher in the animals fed the cholesterol-supplemented diet (1141±18 v. 279±165 mg/dL, p = 0.003), while HDL cholesterol levels were reduced by 37% (42±6 v. 67±5 mg/dL, p = 0.014). There was no difference in plasma triglycerides between the groups (74±30 v. 97±32 mg/dL, p = 0.62).

**Figure 3:**
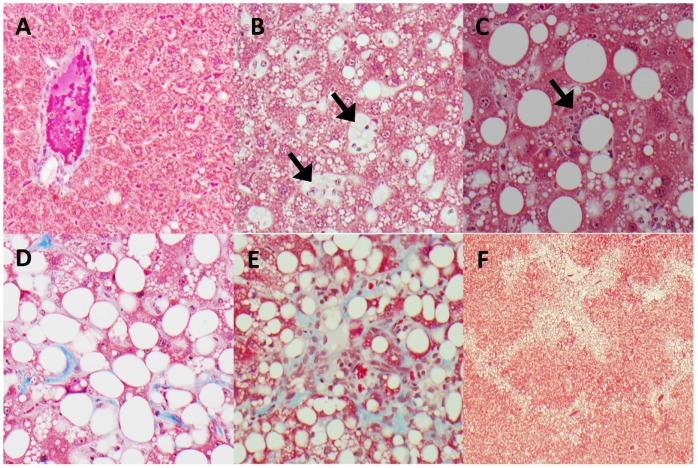
Histopathology of NASH in *P. obesus*. Masson's trichrome stained livers at ×400 magnification except F (x40). A: Control group with minimal steatosis and no fibrosis, B: Lipid droplets in most cells, and sinusoidal foamy macrophages (arrows), C: Parenchymal inflammation with neutrophils (arrow), D: Strands of pericellular and perisinusoidal collagen (arrows), E: Fibrous expansion of portal tracts, with oval cells/ductular reaction, F: Distorted architecture, with portal septum formation.

**Figure 4:**
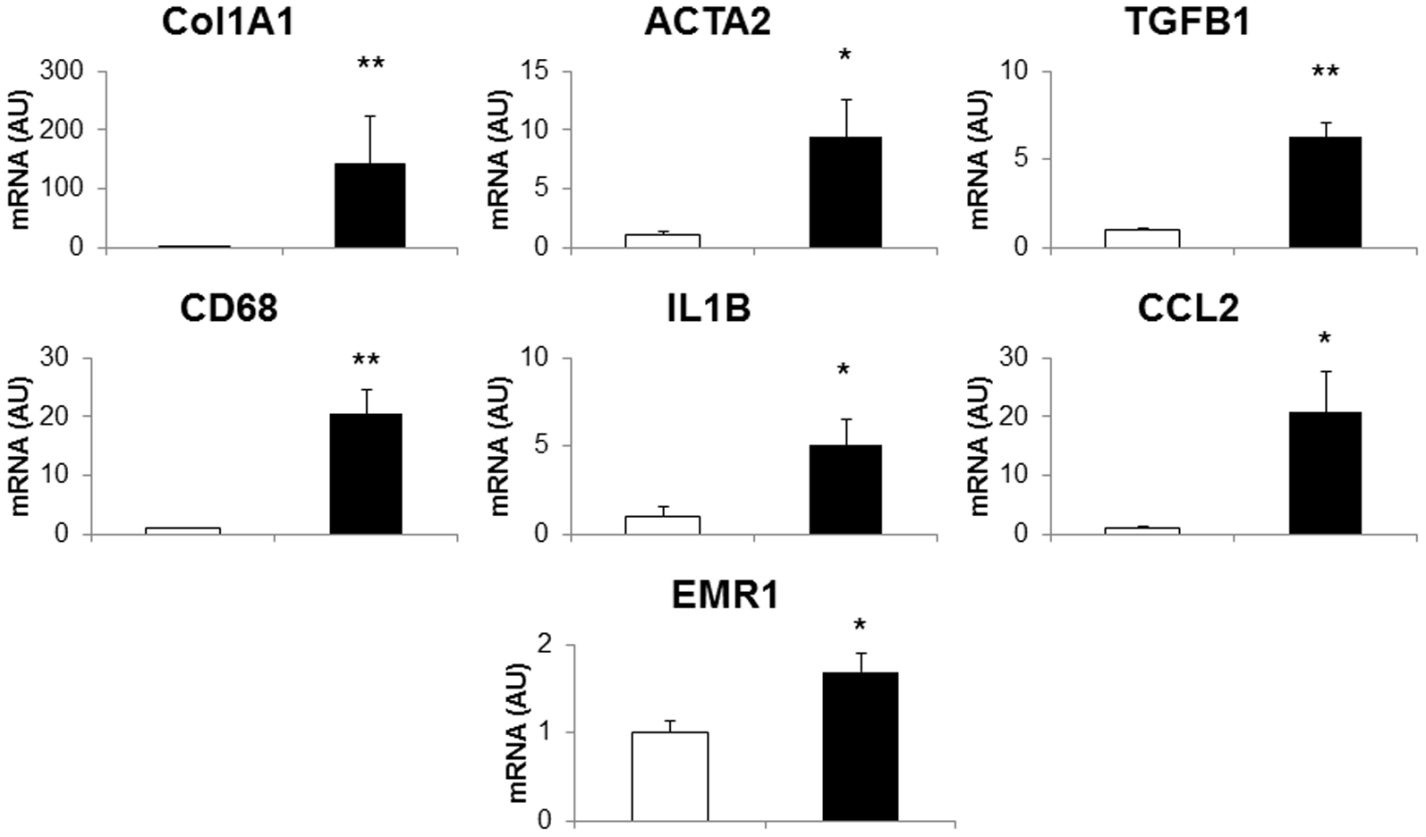
Hepatic gene expression in *P*. *obesus* with NASH. Gene expression of markers of inflammation and fibrosis in livers of *P. obesus* with NASH, n = 8–9 per group. *p<0.05, **p<0.005.

Liver sections from animals on both diets were examined for histological features of NASH (Fig. 3.). Blinded histopathological analysis confirmed the presence of steatosis, inflammation, hepatocyte injury and fibrosis in the cholesterol-supplemented group. Steatosis was marked, with abundant large and small lipid droplets (Fig. 3B,D.). Inflammation was demonstrated by the presence of neutrophil-containing parenchymal inflammatory foci (Fig. 3C.), and in addition sinusoidal foamy macrophages were seen in some livers (Fig. 3B.). This was supported by greater mRNA expression of proinflammatory markers including *CD68, interleukin 1 beta (IL1B), chemokine (C-C motif) ligand 2 (CCL2) and egf-like module containing, mucin-like, hormone receptor-like 1 (EMR1)* (Fig. 4, p<0.05 for each gene). Hepatocyte injury was noted, including focal necrosis in some cases, and there was architectural disruption including fibrous expansion of portal tracts, early septum formation, and both pericellular and perisinusoidal fibrosis (Fig. 3D,E,F.). This fibrosis was reflected by markedly higher *collagen 1 alpha (COL1A1), actin, alpha 2, smooth muscle, aorta (ACTA2) and transforming growth factor, beta 1 (TGFB1)* gene expression (Fig. 4, p<0.05 for each gene). Collagen content was 30-fold increased in the cholesterol-supplemented group compared with controls as assessed by quantitative imaging (11756±5859 vs. 378±224 pixels per field, p<0.002).

NAFLD activity scores were calculated based on the method of Kleiner and Brunt [18]. The overall scores were 4.4±0.5 in the cholesterol-supplemented group and 1.8±0.4 in the control group (p<0.001). Scores were significantly different for steatosis (2.3±0.3 vs. 1.3±0.2, p = 0.007) and inflammation (2.1±0.4 vs. 0.5±0.3, p = 0.003). Histologically, *P. obesus* showed the key features of NASH after only 4 weeks of dietary cholesterol supplementation.

The effect on the liver by simply adding cholesterol to the diet (without increasing dietary fat content) was profound in *P. obesus* compared with effects seen previously in other rodents, suggesting increased susceptibility to NASH in these animals. Therefore, we profiled the hepatic expression of key genes involved in lipid metabolism in *P. obesus* fed the two diets. Expression of genes involved in delivery and uptake of lipids were consistently lower in the cholesterol-supplemented group (*SREBF2* −37%, p = 0.019; *SCARB1* −48%, p = 0.002; *LDLR* −61%, p<0.001; *PCSK9* −50%, p = 0.022; Table 2), while genes involved in fatty acid and triglyceride synthesis were also lower in the cholesterol-supplemented group (*FABP1* −48%, p = 0.012; *PPARA* −59%, p<0.001; *GPAM* −41%, p = 0.016; *LIPE* −49%, p = 0.005; Table 2). In general, genes encoding proteins involved in VLDL synthesis, triglyceride and cholesterol export from the cells were higher in the cholesterol-supplemented group (*ACAT2* +63%, p = 0.034; *ABCA1* +230%, p = 0.001; Table 2), however expression of *MTTP* was unexpectedly lower (−43%, p = 0.007). *PPARD* gene expression was increased (+170%, p = 0.007), presumably to increase transcription of genes involved in fatty acid oxidation, however *CPT1A* mRNA was decreased (−41%, p = 0.027). Finally, the expression of cholesterol 7 alpha hydroxylase (*CYP7A1*), the rate-limiting step in bile acid synthesis, was reduced in livers of *P. obesus* fed the cholesterol-supplemented diet (−47%, p = 0.016).

**Table 2:** Gene expression in livers of *P. obesus* fed the cholesterol-supplemented diet, expressed as fold difference relative to animals fed the standard diet.

*Gene Name*	*Gene Symbol*	*Fold difference*	*P*
Delivery and uptake of lipids			
Sterol regulatory element binding transcription factor 2	SREBF2	0.63±0.07	0.019
Scavenger receptor class B, member 1	SCARB1	0.52±0.08	0.002
Low density lipoprotein receptor	LDLR	0.39±0.04	<0.001
Proprotein convertase subtilisin/kexin type 9	PCSK9	0.58±0.12	0.022
Lipogenesis			
Fatty acid binding protein 1, liver	FABP1	0.52±0.09	0.012
Peroxisome proliferator-activated receptor alpha	PPARA	0.41±0.08	<0.001
Glycerol-3-phosphate acyltransferase, mitochondrial	GPAM	0.59±0.09	0.016
Hormone-sensitive lipase	LIPE	0.51±0.05	0.005
Fatty acid synthase	FASN	0.72±0.06	0.18
Lipid export			
Acetyl-CoA acetyltransferase 2	ACAT2	1.63±0.24	0.034
ATP-binding cassette, sub-family A (ABC1), member 1	ABCA1	3.30±0.49	0.001
Microsomal triglyceride transfer protein	MTTP	0.57±0.08	0.007
Lipid oxidation			
Peroxisome proliferator-activated receptor delta	PPARD	2.70±0.18	0.007
Carnitine palmitoyltransferase 1A (liver)	CPT1A	0.59±0.12	0.027
Bile acid synthesis			
Cholesterol 7 alpha hydroxylase	CYP7A1	0.53±0.10	0.016

## Discussion

Here we have shown that *P. obesus* exhibit key features of NASH when exposed to a cholesterol-supplemented diet. Four weeks of dietary cholesterol supplementation resulted in these animals displaying extensive steatosis, inflammation, hepatocyte injury and fibrosis. These histological phenotypes closely resemble the NASH disease profile observed in human patients. Additionally, hepatic gene expression data confirmed that the NASH in *P. obesus* appears to be mechanistically similar to that in patients with NASH.

Brunt and colleagues [4], [5] have established criteria for the histological diagnosis of NASH. The key features are steatosis, hepatocyte injury and inflammation, typically localised in zone 3, with or without fibrosis. In this study we have shown the presence of all of these key features of NASH in livers of *P. obesus* fed a cholesterol-supplemented diet for 4 weeks. The steatosis was closely associated with foci of inflammation, and was particularly evident in hepatic zone 3. Our evidence for hepatocyte injury were less clear, although the histological appearance suggested significant disruption of the cellular architecture, and this was supported by increased plasma levels of transaminases that are known to be released from injured hepatocytes. Furthermore, patches of necrosis observed in two of the animals also support the suggestion of hepatocyte injury, even though necrosis is not a common feature of human NASH [4], [5]. Overall, the histological appearance of NASH in *P. obesus* closely resembles that seen in human patients.

In contrast to other dietary based animal models, the diet used in this study involved only a minor modification to a standard rodent diet. Other dietary models are either based on nutrient deficiency (e.g. MCD diet) or unrealistically high energy diet (e.g. 60–80% kcal/fat, 80% fructose) [7], [8]. In this study we added 2% cholesterol to a standard rodent diet, from which 20% of energy was derived from fat, which is not considered to be a high fat diet. The addition of 2% cholesterol to the diet can be considered relatively high when compared with previous studies using 0.5–1% dietary cholesterol [19], [20]. However, more recently, 2% dietary cholesterol in the context of high fat, high fructose diet (where the source of fat was *trans*-fat) elicited fibrosis in leptin-deficient mice, but not in normal C57BL6 mice [21], suggesting that multiple insults are required in other rodent models for the full spectrum of NASH, not just increased dietary cholesterol [22]. The presence of significant hepatic damage induced by relatively minor dietary variation suggests that *P. obesus* may be an attractive model for future NASH research compared with other dietary-based models.

In this study we have shown the development of NASH in *P. obesus* occurs after only four weeks. Such rapid onset is not observed in other dietary models with exposure to MCD diet requiring up to 10 weeks to lead to the development of NASH (depending on species/strain), whilst exposure to a high fat diet, fructose diet, or an atherogenic diet can take up to several months before NASH is observed [7], [8]. We note that the animals in this study were not obese. It will be of interest to repeat this study in animals with pre-existing obesity and/or type 2 diabetes and investigate how this affects the development of NASH.

In *P. obesus* fed the cholesterol-supplemented diet for 4 weeks there was a consistent reduction in the liver mRNA levels of genes encoding proteins involved in the delivery and uptake of lipids. Of note, LDL receptor (LDLR) mRNA was 61% lower in animals fed the cholesterol-supplemented diet, which would be expected to result in reduced lipid and cholesterol uptake by the cells. This is opposite to what has been observed in mouse models of NASH [23], but consistent with observations in African Green Monkeys with NASH [24]. We also observed reductions in genes encoding proteins involved in de novo fatty acid and triglyceride synthesis. Notably, fatty acid binding protein 1 mRNA was significantly reduced in *P. obesus* with NASH, as it is in human cases [25], but not in mouse models of NASH [26]. Gene expression of PPARA and hormone sensitive lipase (*LIPE*) were reduced in *P. obesus* with NASH, as they are in humans with NASH [27], [28].

Hepatic gene expression of ACAT2, which is involved in the removal of cholesterol from hepatocytes via VLDL, and ABCA1, which is involved in direct cholesterol efflux from hepatocytes, were both significantly increased in *P. obesus* with NASH. Increased ACAT2 gene expression is also a feature of human NASH [29]. Gene expression of MTTP was unexpectedly reduced, as has been seen in various mouse models of NASH [30], [31]. CPT1A gene expression was also unexpectedly reduced, similar to what has been observed in human NASH [28], [32], possibly due to excessive accumulation of malonyl-CoA in advanced NASH. Finally, the gene expression of cholesterol 7 alpha hydroxylase (CYP7A1), the rate limiting step in bile acid synthesis, was reduced by 47% (p = 0.02) in livers of *P. obesus* with NASH. This is counterintuitive, as bile acid synthesis is a major path for removal of cholesterol from the liver. However a similar result was also found in monkeys with NASH [24], and is thought to represent a measure to reduce intestinal cholesterol absorption by limiting bile acid availability, in an attempt to restore hepatic cholesterol homeostasis.

In summary we observed a general decrease in the expression of genes encoding proteins involved in the uptake and synthesis of fatty acids and triglycerides in the liver, and an increase in the expression of genes encoding proteins that function in the export or oxidation of lipids. Collectively these data indicate that the hepatic molecular characteristics of NASH in *P. obesus* resemble those seen in the human disease.

*P. obesus* rapidly develop NASH when fed a cholesterol-supplemented diet including marked steatosis, inflammation, hepatocyte injury and fibrosis. In general, the gene expression patterns and biochemical and histological features observed in *P. obesus* with NASH closely resembled the human condition. We conclude that *P. obesus* is a new animal model of NASH with significant advantages over existing models.
